# Expansion of mixed immune cells using CD3/CD161 co-stimulation for the treatment of cancer

**DOI:** 10.1038/s41598-023-33987-2

**Published:** 2023-04-26

**Authors:** Ryo Tsumura, Miwa Haruta, Masataka Kuwano, Masahiro Yasunaga

**Affiliations:** 1 Division of Developmental Therapeutics, EPOC, National Cancer Center, 6-5-1, Kashiwanoha, Kashiwa, Chiba 277-8577 Japan; 2 Dojin institute of cancer immunology, Co., Ltd. Kumamoto, Kumamoto, 862-0967 Japan

**Keywords:** Biological techniques, Cancer, Immunology, Oncology

## Abstract

Adoptive cell transfer (ACT) is a type of personalized immunotherapy in which expanded immune cells are administered to patients with cancer. However, single-cell populations, such as killer T cells, dendritic cells, natural killer (NK) cells, and NKT (NKT) cells, have been generally used, and their effectiveness remains limited. Here, we established a novel culture method via CD3/CD161 co-stimulation and successfully expanded CD3^+^/CD4^+^ helper T cells, CD3^+^/CD8^+^ cytotoxic T cells (CTLs), CD3^−^/CD56^+^ NK cells, CD3^+^/CD1d^+^ NKT cells, CD3^+^/CD56^+^ NKT cells, CD3^+^/TCRγδ^+^ T cells, and CD3^−^/CD11c^+^/HLA-DR^+^ dendritic cells in peripheral blood mononuclear cells from healthy donors; their respective numbers were 155.5, 1132.5, 5.7, 117.0, 659.2, 325.6, and 6.8 times higher than those before expansion. These mixed immune cells showed strong cytotoxicity against cancer cell lines Capan-1 and SW480. Moreover, both CD3^+^/CD8^+^ CTLs and CD3^+^/CD56^+^ NKT cells killed tumor cells in cell contact-dependent and -independent manners via granzyme B and interferon-γ/TNF-α, respectively. Furthermore, the cytotoxicity of the mixed cells was significantly superior to that of CTLs or NKTs alone. A bet-hedging CTL-NKT circuitry is one potential mechanism underlying this cooperative cytotoxicity. Collectively, CD3/CD161 co-stimulation may be a promising culture method to expand multiple, distinct immune cell populations for the treatment of cancer.

## Introduction

Over the past half century, several cancer immunotherapies have been developed, one of which is adoptive cell transfer (ACT). This treatment involves the systemic administration of peripheral blood-derived immune cells or tumor-infiltrating lymphocytes (TILs). These immune cells are obtained from the patient and then expanded ex vivo. ACT therapy has many advantages over other immunotherapies, including the artificial expansion of a sufficient number of immune cells with the functions necessary to exert antitumor effects. In addition, in vitro culture manipulation allows these immune cells to be effectively activated by freeing them from the effects of various immunosuppressive factors that exist in vivo. Interleukin-2 (IL-2) is a T-cell growth factor that has been frequently used to induce both the proliferation and activation of T cells^1^. One study reported that the intravenous injection of immune lymphocytes expanded with IL-2 was effective in an animal tumor model^2^. Other studies subsequently examined the infusion of autologous lymphokine-activated killer (LAK) cells^3^ and TILs^4^ in patients with several types of cancer, including metastatic melanoma. A major contributor to the antitumor activity of these LAK cells is the significant expansion of CD4^+^ T cells and CD8^+^ T cells^5^. Also, populations of TILs that arise in tumors generally consist of CD4^+^ T cells and CD8^+^ T cells^6,7^. These effector T cells can be highly activated and expanded in vitro with IL-2 and anti-CD3 and anti-CD28 monoclonal antibodies (mAbs)^6,8^. In recent years, autologous T cells from the peripheral blood of patients with cancer have been genetically engineered to express antitumor T-cell receptors (TCRs) or chimeric antigen receptors (CARs) and thus enhance their tumor cell cytotoxic activity^9^.

In addition to these major T-cell populations, invariant natural killer T (iNKT) cells have been investigated in ACT therapy^10–14^. iNKT cells constitute a CD1d-restricted T-cell subset that expresses an invariant TCRα chain (Vα24-Jα18) paired with a single TCRβ chains (Vβ11)^15,16^. Because iNKT cells play important roles in anti-tumor immunity, including direct and indirect cytotoxicity, memory formation, and inhibition of immune suppressive cells^17^, their administration is a promising cancer treatment strategy. In addition to the aforementioned T-cell populations, natural killer (NK) cells have been evaluated regarding their utility in ACT therapy^18^. NK cells are effector immune cells that possess strong cytotoxicity^19^, and because they recognize targeted cancer cells without requiring interaction between a T-cell receptor (TCR) and human leukocyte antigen (HLA), their injection does not induce graft-versus host disease^20^. Furthermore, NK cells can selectively attack cancer cells that express relatively low levels of self-major histocompatibility complex (MHC) class I molecules that inhibit NK-cell activity^21^. Interestingly, NK cells exhibit enhanced antitumor activity when they are near CD8^+^ T cells^22^. Therefore, it has been hypothesized that a bet-hedging strategy involving a variety of immune cells might eradicate tumor cells most effectively.

However, previous studies of ACT therapy have mainly focused on the administration of one or two immune cell populations, such as CD3^+^/CD8^+^ T cells, CD3^+^/CD4^+^ T cells, NK T (NKT) cells, and NK cells, and their in vivo effectiveness has been limited. One reason may be the lack of a coordinated immune network in which multiple types of immune cell act cooperatively against tumor cells. In contrast, two types of mixed immune cells generated from peripheral blood mononuclear cells (PBMCs) have been reported. The first type consists of cytokine-induced killer (CIK) cells that are cultured with anti-CD3 mAb and IL-2 after the induction of IFN-γ, and include mixed effector cells such as CD3^+^/CD56^+^ NKT cells, CD3^+^/CD8^+^ T cells, CD3^+^/CD4^+^ T cells, and CD3^−^/CD56^+^ NK cells^23^. The second type comprises mixed immune cells, specifically Vα24^+^/Vβ11^+^ iNKT cells, CD3^+^ T cells, and CD3^−^/CD56^+^ NK cells that are induced by the αGalCer-pulsed IL-2/GM-CSF culture method^24^. Immune networks could be coordinated in these mixed immune cells, which may facilitate their cooperative and effective killing of cancer cells. In the present study, we propose a novel culture method for ACT therapy consisting of CD3/CD161 co-stimulation with IL-2 and granulocyte-colony stimulating factor, with the goal of expanding multiple distinct immune cell populations from a donor’s PBMCs. CD161 is a C-type lectin-like receptor expressed on the majority of NK cells and on some T cells, such as NKT cells, CD4^+^ T cells, and CD8^+^ T cells^25^. CD161 expressed on NK cells has been well investigated and has been shown to function as an inhibitory receptor^26^. In NKT cells, however, Exkey et al. reported that proliferation and cytokine secretion were augmented by CD3/CD161 co-stimulation rather than CD3 or CD161 mono-stimulation^27^. Here, we successfully expanded PBMCs to a large number of mixed immune cells. Interestingly, this immune cell combination showed greater cytotoxicity than two individual fractions, namely CD3^+^/CD8^+^ CTLs and CD3^+^/CD56^+^ NKT cells. We suggest that CD3/CD161 co-stimulation is a useful culture method to expand multiple, distinct immune cell populations for the treatment of cancer.

## Materials and methods

### Cell cultures

Capan-1, AsPC-1 (a pancreatic cancer cell line) and SW480 (a colorectal cancer cell line) cells were obtained from the American Type Culture Collection (ATCC, Manassas, VA, USA). These cell lines were cultured in ATCC-recommended medium supplemented with fetal bovine serum (FBS, Thermo Fisher Scientific, Waltham, MA, USA), penicillin G (100 units/mL), streptomycin (100 μg/mL), and amphotericin B (0.25 μg/mL, FUJIFILM Wako Pure Chemical Corporation, Osaka, Japan) in a 5% CO_2_ atmosphere at 37 °C. PBMCs from three uncharacterized healthy donors (Lot.HHU20180419, Lot.HHU20180710, and Lot.HHU20200303) were obtained from Cellular Technology Limited (Shaker Heights, OH, USA). PBMCs were cultured with RPMI-1640-based medium containing 1 ~ 5% Sodium Heparin Human Plasma (ViroQuest, Osaka, Japan), 1000 U/mL human interleukin-2 (hIL-2), and 0-1013 U/mL human granulocyte colony-stimulating factor (hG-CSF) in a culture flask pre-coated with anti-CD3 mAb (1 µg/mL, UCTH-1, TONBO Biosciences, San Diego, CA, USA) and anti-CD161-PE-conjugated mAb (10 µg/mL, 191B8, Immunotech, Marseille, France) for 24 h at room temperature. The mixed immune cells were frozen at − 80 °C until use. Because of an HLA-match with Capan-1 and SW480 cells, Lot.HHU20180419 (HLA-A*24:02-positive) was used for subsequent experiments, except for the analysis of immune cell numbers.

### Cell-surface staining by FCM analysis

Cells derived from three different donors were suspended in Dulbecco’s PBS (DPBS, Thermo Fisher Scientific) with 0.5% bovine serum albumin and 2 mM EDTA (B.E.PBS). After blocking with FcR blocking regent (Miltenyi Biotec, Bergisch Gladbach, Germany) at 4 °C for 10 min, the cells were stained with the following mAbs to identify specific cell populations: anti-CD3 mAb AlexaFluor 488 (Biolegend, San Diego, CA, USA), anti-CD3 mAb PerCP (Biolegend), anti-CD4 mAb APC (Biolegend), anti-CD8 mAb APC (Biolegend), anti-CD56 mAb APC (Biolegend), anti-TCRγδ mAb APC (Biolegend), anti-CD11c mAb FITC (Biolegend), anti-HLA-DR mAb APC (Biolegend), and CD1d Tetramer APC (α-GalCer loaded, Medical & Biological Laboratories Co., Ltd., Tokyo, Japan). Then, the cells were washed twice with B.E.PBS and fixed with 4% paraformaldehyde (PFA) in DPBS for 15 min at room temperature. The fluorescence intensity of the cells was measured using a Guava EacyCyte flow cytometer (Merck Millipore, Burlington, MA, USA) and analyzed by FlowJo analysis software (Tree Star Inc, Ashland, OR, USA). In the present study, dendritic cells were identified by the following combination of cell surface markers: CD3^−^/CD11c^+^/HLA-DR^+^^28,29^. To determine the HLA-A24 expression of cancer cell lines, anti-human HLA-A24 mAb-FITC (Medical & Biological Laboratories co., LTD.) was used.

### Cell-killing assays

Capan-1 and SW480 cells were harvested on 96-well plates (Corning, Corning, NY, USA) and incubated overnight. To investigate the direct killing activity of mixed immune cells, these cells were applied to wells and co-cultured with cancer cells at various effector-to-tumor (E:T) ratios for 72 h. To assess indirect killing, the mixed immune cells were cultured for 48 h at 5,000,000 cells/mL, and their supernatants were collected. Supernatant diluted by 8 or 32 fold was applied to the cancer cells and incubated for 72 h. Cell viabilities were determined using a Cell Counting Kit-8 (Dojindo Laboratories, Kumamoto, Japan) in all assays.

### Immunofluorescence staining

Capan-1 and SW480 cells were seeded on Falcon 4-well culture slides and incubated overnight. The mixed immune cells were added (E:T ratio = 10:1) and co-cultured with cancer cells for 6 h. After fixation with 4% PFA in DPBS for 15 min at room temperature, non-specific binding of antibodies was blocked with 5% skim milk in DPBS for 1 h at room temperature. Then, cells were stained with cetuximab (Merck, Kenilworth, NJ, USA), rat anti-CD3 mAb (Abcam, Cambridge, UK), and rabbit anti-granzyme B mAb (Cell Signaling Technology, Danvers, MA, USA). Goat anti-human polyclonal antibody (pAb) AlexaFluor 555 (Thermo Fisher Scientific), goat anti-rat pAb AlexaFluor 647 (Thermo Fisher Scientific), and goat anti-rabbit pAb AlexaFluor 488 (Thermo Fisher Scientific) were used as secondary antibodies. Cell nuclei were stained with DAPI solution (Thermo Fisher Scientific). All fluorescence images were obtained using a BZ-X710 fluorescence microscope (Keyence, Osaka, Japan).

### Intracellular staining by FCM analysis

The FCM procedure is described above. Prior to staining, cells were activated by treatment with reagents in the BD Cytofix/Cytoperm Plus Fixation/Permeabilization Kit with BD GolgiPlug Protein Transport Inhibitor (Becton Dickinson and Company, Franklin Lakes, NJ, USA), then treated with fixation and permeabilization solutions. Anti-CD3 mAb PerCP, anti-CD8 mAb APC, anti-CD56 mAb APC, anti-IFN-γ mAb FITC (Biolegend), anti-TNF-α mAb AlexaFluor 488 (Biolegend), and rabbit anti-granzyme B mAb were used to identify effector T cells and to evaluate the expression of intracellular proteins. Isotype IgG1κ FITC (Biolegend), isotype IgG1κ AlexaFluor 488 (Biolegend), and rabbit IgG Isotype Control (Thermo Fisher Scientific) were used as isotype control antibodies. Goat anti-rabbit pAb AlexaFluor 488 was used as a secondary mAb.

### Cell sorting

The cells were stained using the FCM analysis procedure using anti-CD3 mAb AlexaFluor 488, anti-CD8 mAb APC, and anti-CD56 mAb APC. Then, the CD3^+^/CD8^+^ and CD3^+^/CD56^+^ populations were sorted using an SH800H cell sorter (Sony, Tokyo, Japan). Cell purification was confirmed using a Guava EacyCyte flow cytometer. After cell sorting, each cell population was cultured for 3 days in proper conditions. The number and viability of sorted cells were evaluated using trypan blue staining prior to performing the cell killing assay.

### Statistical analysis

All data are shown as the mean ± standard deviation (SD). For the evaluation of cytotoxicity, statistically significant differences between more than three groups were determined by one-way ANOVA with Tukey analysis. Statistical analysis was performed using EZR software.

## Results

### Novel culture method and immune cell expansion

**Figure 1:**
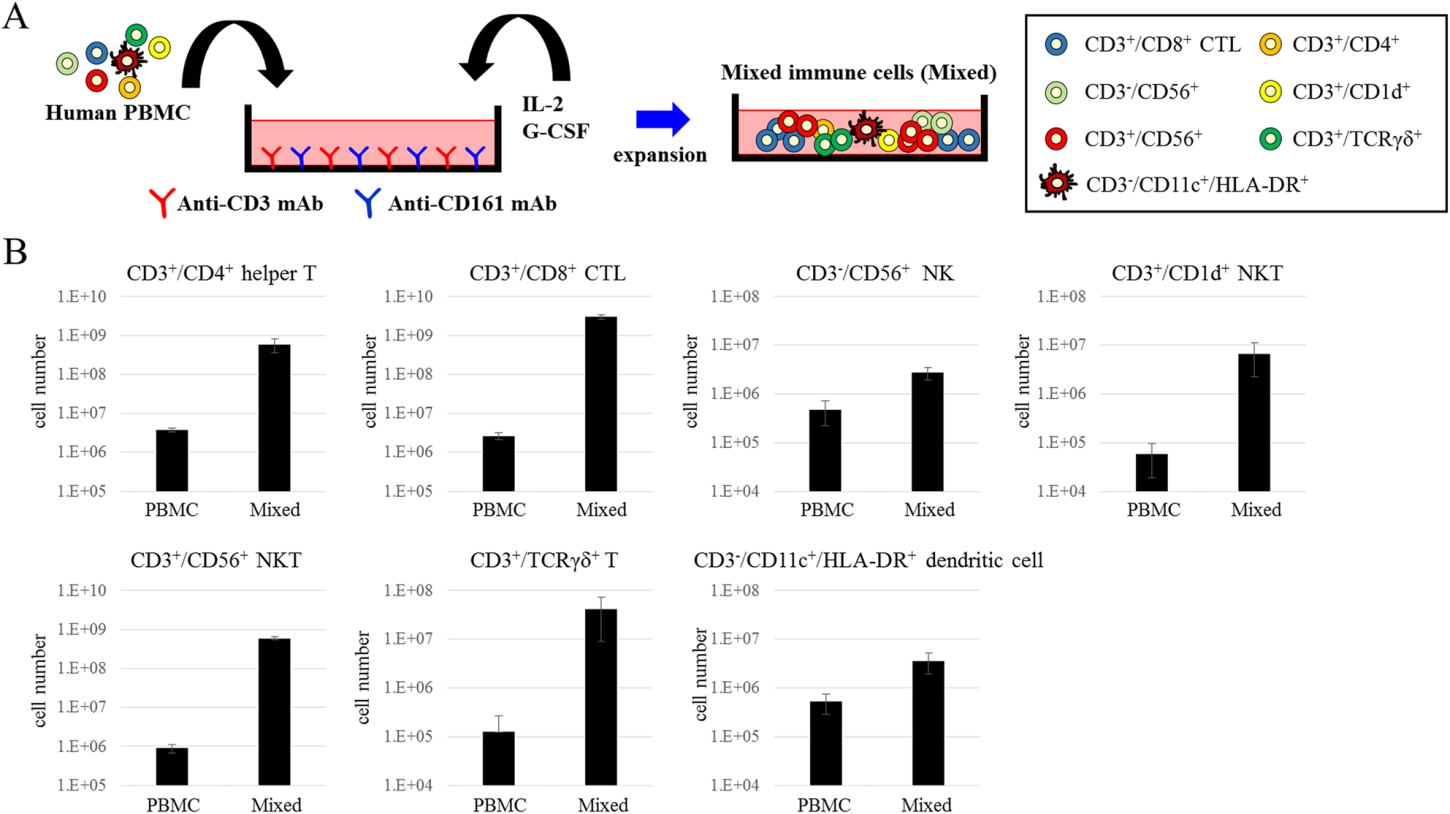
Novel culture method and expansion of immune cells. (**A**) Diagram of the novel culture method, consisting of CD3/CD161 co-stimulation with IL2 and G-CSF. (**B**) The numbers of CD3^+^/CD4^+^ helper T cells, CD3^+^/CD8^+^ CTLs, CD3^−^/CD56^+^ NK cells, CD3^+^/CD1d^+^ NKT cells, CD3^+^/CD56^+^ NKT cells, CD3^+^/TCRγδ^+^ T cells, and CD3^−^/CD11c^+^/HLA-DR^+^ dendritic cells before (PBMCs) and after (Mixed) expansion. The experiment was performed using PBMCs and cultured immune cells from three different donors. The data are shown as means ± SD.

We established a novel culture method consisting of CD3/CD161 co-stimulation with IL2 and G-CSF to expand mixed immune cells, including CD3^+^/CD4^+^ helper T cells, CD3^+^/CD8^+^ CTLs, CD3^−^/CD56^+^ NK cells, CD3^+^/CD1d^+^ NKT cells, CD3^+^/CD56^+^ NKT cells, CD3^+^/TCRγδ^+^ T cells, and CD3^−^/CD11c^+^/HLA-DR^+^ dendritic cells (Fig. 1A). With this method, three PBMC lots from healthy volunteers were cultured for 3 weeks and expanded to obtain the mixed immune cells. After expansion, the total cell number in each of the PBMC lots, namely Lot.HHU20180419, Lot.HHU20180710, and Lot.HHU20200303, was increased 350-, 320-, and 370-fold, respectively. We performed FCM analysis to investigate the expansion of the aforementioned immune cell populations, and found that the numbers of CD3^+^/CD4^+^ helper T cells, CD3^+^/CD8^+^ CTLs, CD3^−^/CD56^+^ NK cells, CD3^+^/CD1d^+^ NKT cells, CD3^+^/CD56^+^ NKT cells, CD3^+^/TCRγδ^+^ T cells, and CD3^-^/CD11c^+^/HLA-DR^+^ dendritic cells were 155.5, 1132.5, 5.7, 117.0, 659.2, 325.6, and 6.8 times larger than before expansion, respectively (Fig. 1B). Therefore, this novel culture method successfully expanded these mixed immune cells. The dominant populations of cytotoxic effector cells were CD3^+^/CD8^+^ CTLs and CD3^+^/CD56^+^ NKT cells.

### Cell-killing activity

**Figure 2:**
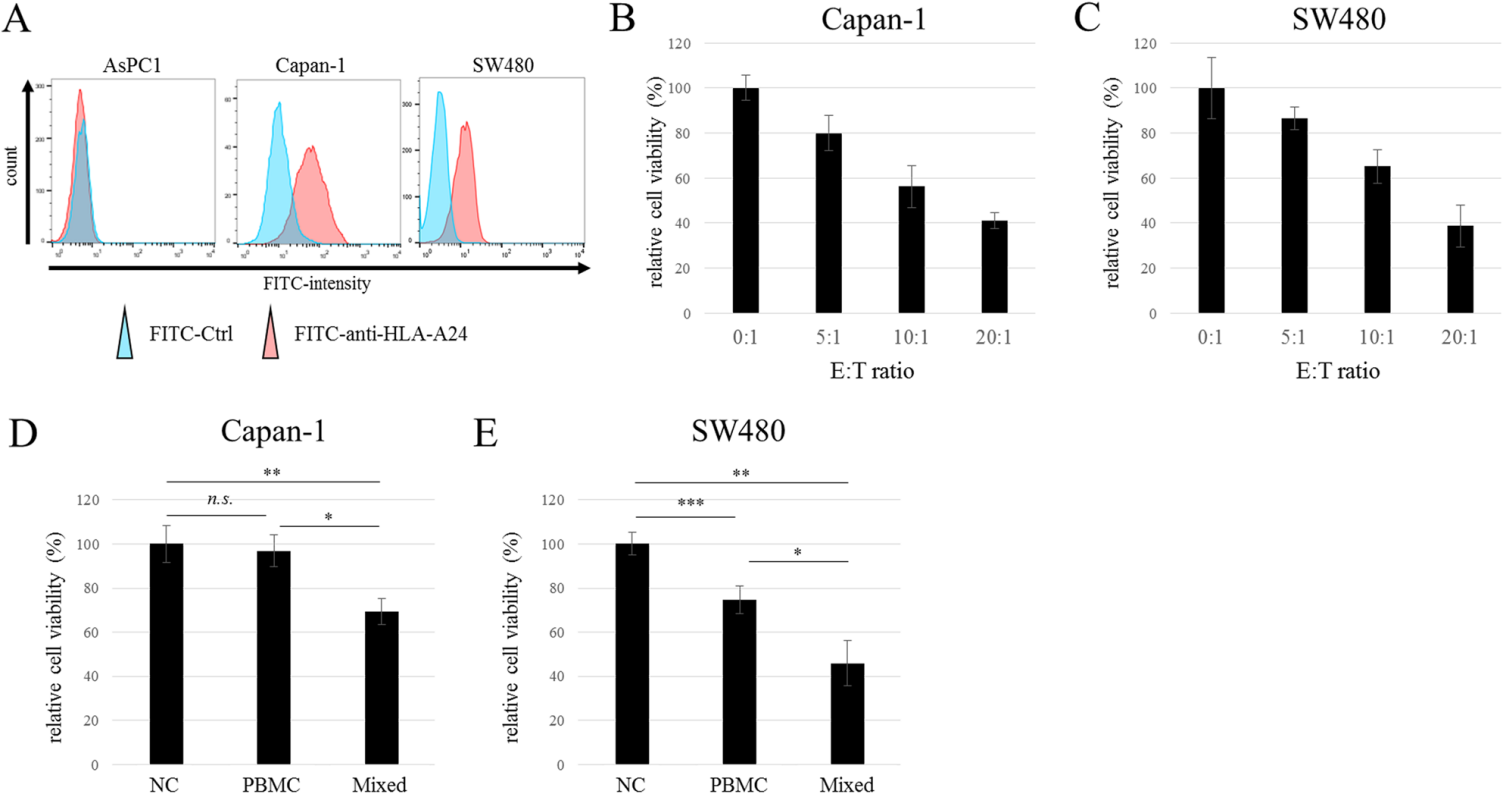
Cytotoxicity in vitro. Flow cytometry analysis of cancer cell lines using HLA-A24 specific antibody (**A**). Cultured immune cells exhibited cell-killing activity against (**B**) Capan-1 and (**C**) SW480 cells that depended on the E:T ratio (*n* = 5). Cytotoxicity of PBMCs and mixed immune cells against (**D**) Capan-1 and (**E**) SW480 cells (E:T ratio = 10:1, *n* = 5). The data are shown as means ± SD. *n.s*. not significant. *p < 0.0005, **p < 0.0001, ***p < 0.001 (one-way ANOVA with Tukey analysis).

**Figure 3:**
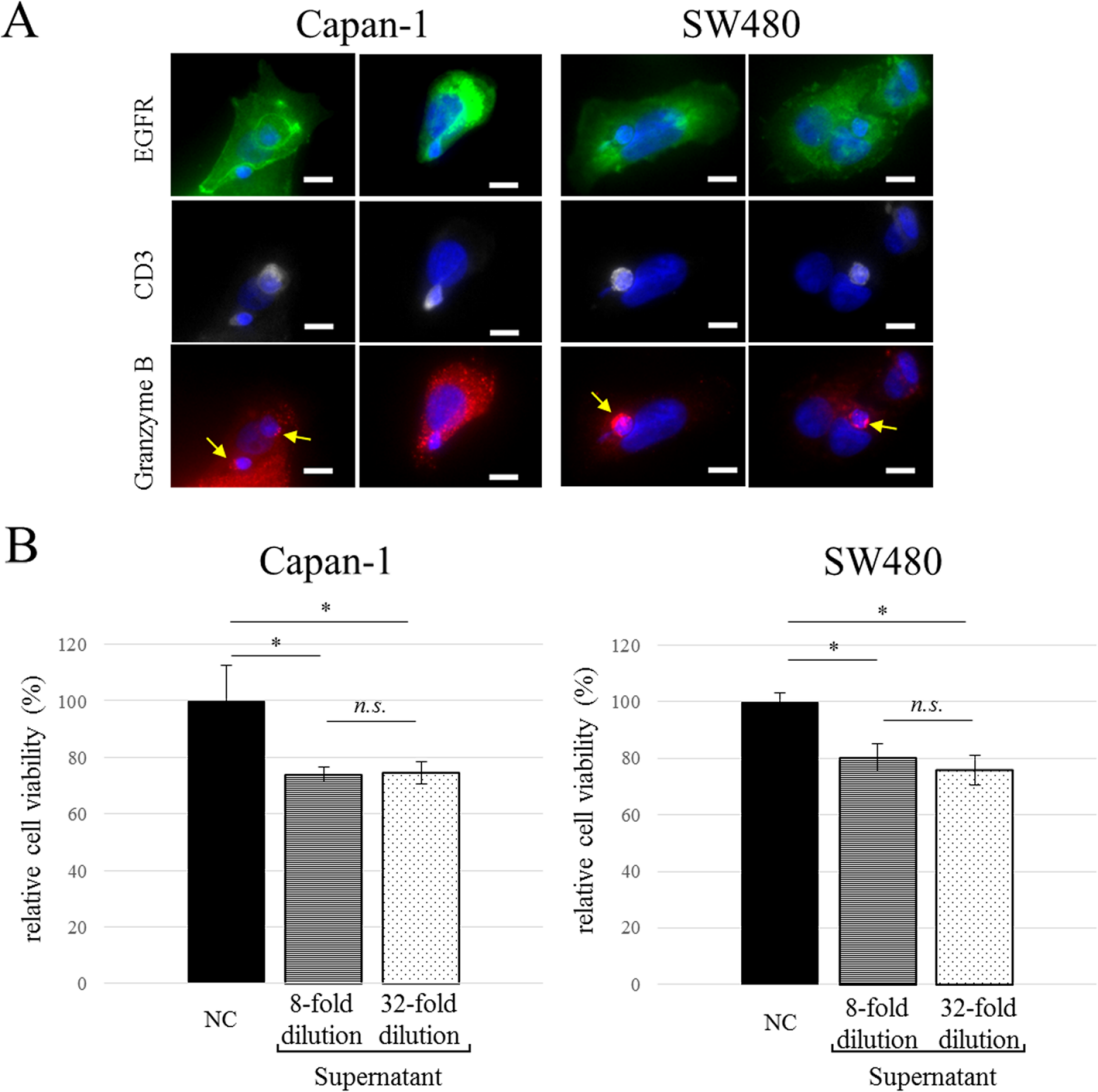
Direct and indirect tumor cell killing. (**A**) Two representative images of granzyme B production by CD3^+^ T cells in contact with Capan-1 or SW480 cells. DAPI, blue; EGFR, green; CD3, white; granzyme B, red. Arrow (yellow) indicates granzyme B expression in CD3^+^ T cells. Scale bar = 10 µm. (**B**) Cell-killing activity of the supernatant of mixed immune cells against Capan-1 and SW480 cells (*n* = 3). The data are shown as means ± SD. *n.s*. not significant. *p < 0.005 (one-way ANOVA with Tukey analysis).

We evaluated the cell-killing activity of the mixed immune cells against cancer cell lines Capan-1 and SW480. Both Capan-1 and SW480 cells were HLA-A24 positive (Fig. 2A). In addition, among three PBMC lots, Lot.HHU20180419 was used because only it was HLA-A24 positive. The immune cells induced cell damage in both cancer cell lines in an E:T ratio-dependent manner (Fig. 2B,C). Although the original PBMCs did not exhibit cytotoxicity against Capan-1 cells, the mixed immune cells showed significant efficacy (Fig. 2D, *p = 0.000064, **p = 0.00018). While the original PBMCs demonstrated cytotoxicity against SW480 (Fig. 2E, ***p = 0.00054), the mixed immune cells were significantly more effective (Fig. 2E, *p = 0.00016, **p = 0.0000003). We next investigated the mechanisms of action (MOA) underlying the cell-killing activity of the mixed immune cells. There are two types of MOAs, cell contact-dependent and -independent (direct and indirect, respectively). Regarding the former, immune fluorescence staining demonstrated that CD3^+^ immune cells directly contacted EGFR-positive Capan-1 and SW480 cancer cells (Fig. 3A). Additionally, CD3^+^ immune cells expressed granzyme B in the cytoplasm, and Capan-1 cells endogenously expressed granzyme B without cell–cell contact (Fig. 3A). Next, we evaluated cell contact-independent tumor cell killing. The 8- and 32-fold diluted supernatant of the mixed immune cells showed cytotoxicity against Capan-1 and SW480 cells, while the negative control did not (Fig. 3B). These results indicated that the cytotoxicity of the mixed immune cells was caused by both cell contact-dependent and -independent mechanisms.

### Production of cytokines and granzyme B

**Figure 4:**
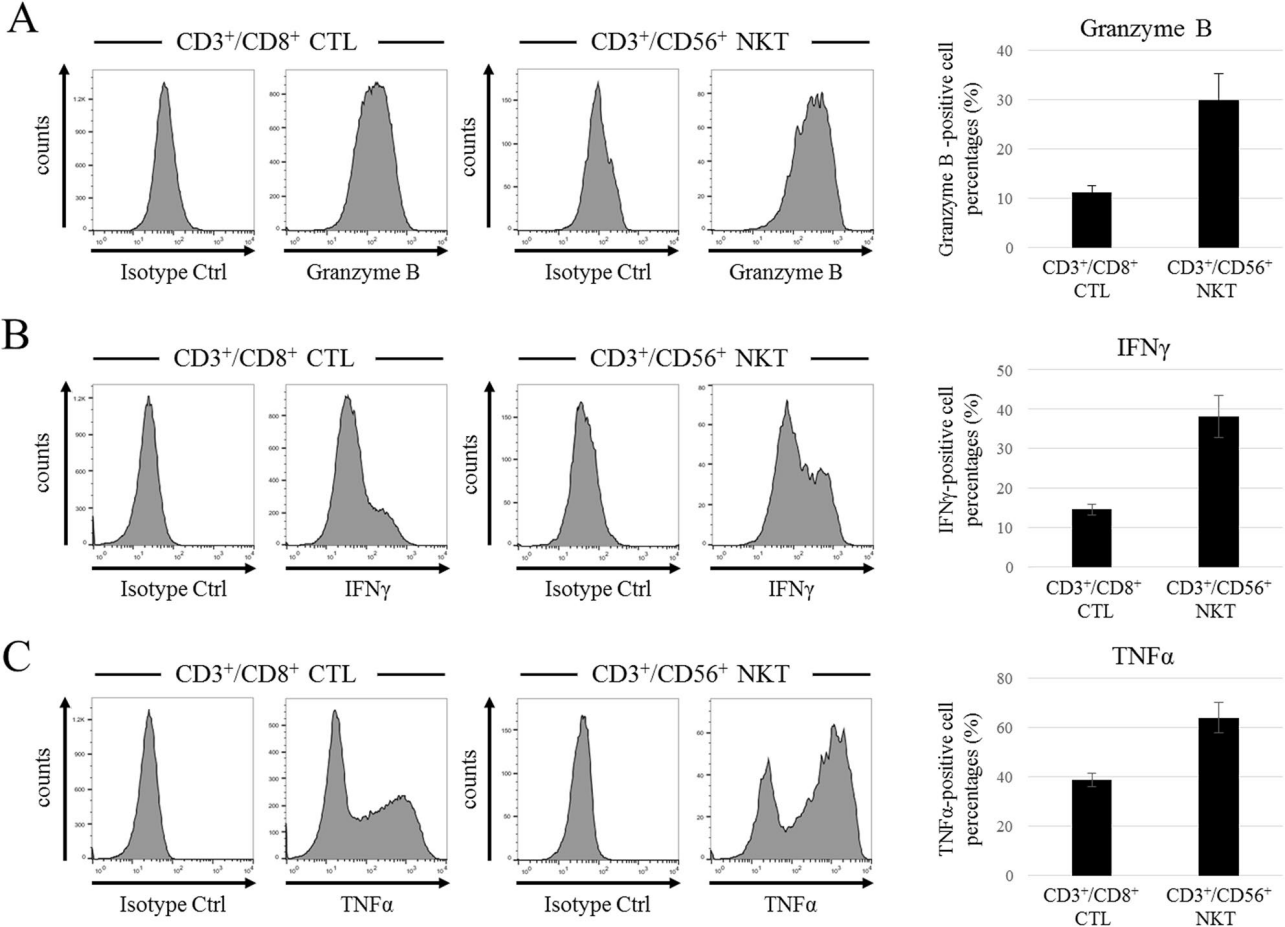
Production of granzyme B and cytotoxic cytokines by CD3^+^/CD8^+^ CTL and CD3^+^/CD56^+^ NKT cell populations in the mixed immune cells. (**A**) FCM histogram data of granzyme B production by CD3^+^/CD8^+^ CTLs and CD3^+^/CD56^+^ NKT cells, and the percentages of granzyme B-positive cells in each population (*n* = 3). (**B**) FCM histogram data of IFN-γ production by CD3^+^/CD8^+^ CTLs and CD3^+^/CD56^+^ NKT cells, and the percentages of IFN-γ-positive cells in each population (*n* = 3). (**C**) FCM histogram data of TNF-α production by CD3^+^/CD8^+^ CTLs and CD3^+^/CD56^+^ NKT cells, and the percentages of TNF-α-positive cells in each population (*n* = 3). The data are shown as means ± SD.

CD3^+^/CD8^+^ CTLs and CD3^+^/CD56^+^ NKT cells were the major populations in mixed immune cells and both were considered to be cytotoxic effector cells. Therefore, we compared the MOAs of these cell types to determine which contributed more significantly to cytotoxicity. We examined the expression of granzyme B, interferon (IFN)-γ, and tumor necrosis factor (TNF)-α all of which play a major role in cell contact-independent tumor cell killing. FCM analysis showed that granzyme B expression in CD3^+^/CD56^+^ NKT cells was higher than that in CD3^+^/CD8^+^ CTLs (Fig. 4A). Similarly, although IFN-γ and TNF-α were strongly expressed in both CD3^+^/CD8^+^ CTLs and CD3^+^/CD56^+^ NKT cells, their expression was greater in CD3^+^/CD56^+^ NKT cells (Fig. 4B,C). These results indicate that CD3^+^/CD8^+^ CTLs and CD3^+^/CD56^+^ NKT cells kill tumor cells efficiently in both a cell contact-dependent and -independent manner.

### Comparing the cytotoxicity of mixed immune cells and single-cell populations

**Figure 5:**
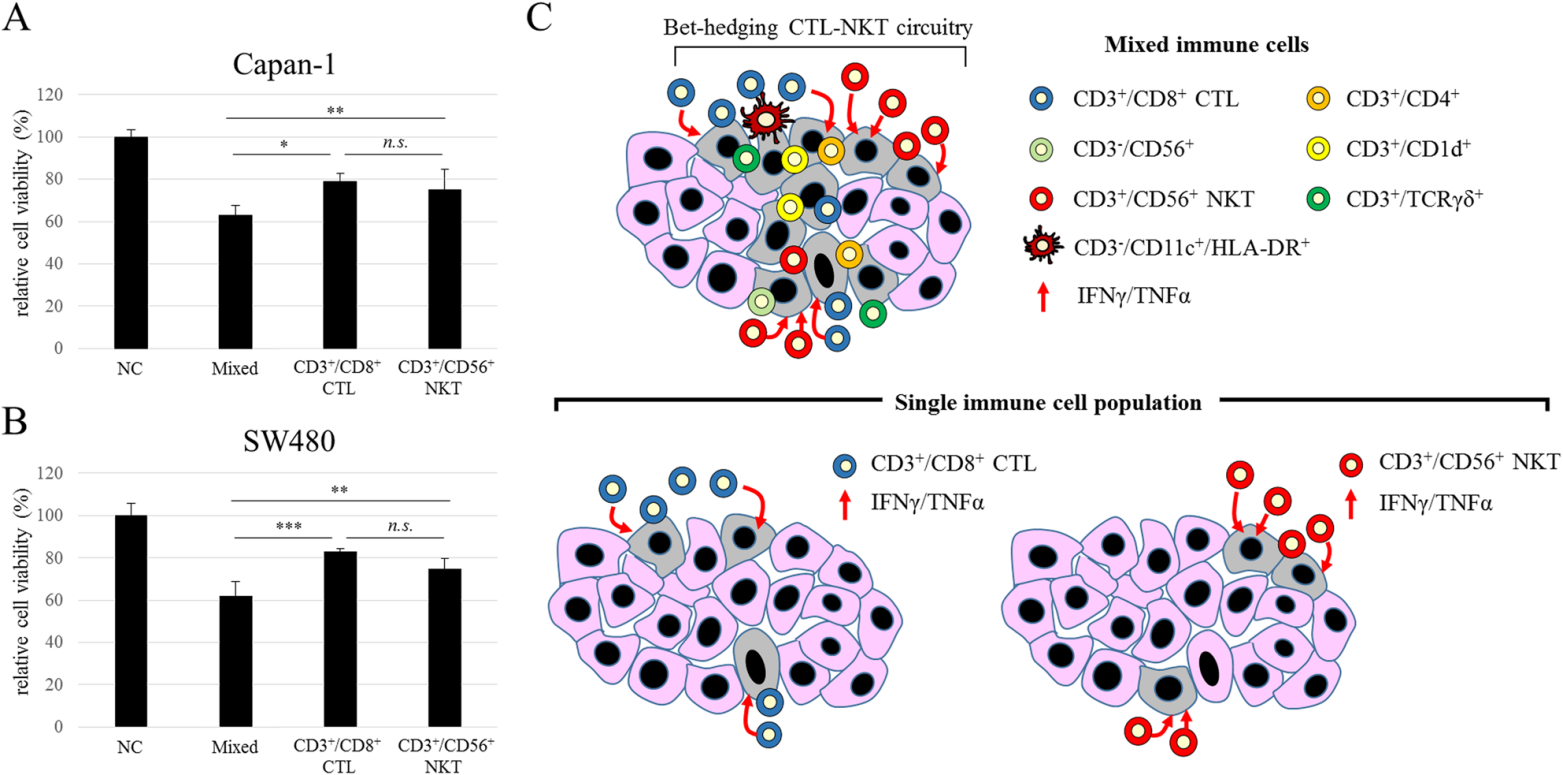
Comparison of cytotoxicity of mixed immune cells and single-cell populations. Cytotoxicity of mixed immune cells and single cell populations of CD3^+^/CD8^+^ CTLs and CD3^+^/CD56^+^ NKT cells against (**A**) Capan-1 and (**B**) SW480 cells (E:T ratio = 10:1, *n* = 4). The data are shown as means ± SD. *n.s*. not significant. *p < 0.01, **p < 0.05, ***p < 0.0005 (one-way ANOVA with Tukey analysis). (**C**) Diagram of tumor cell-killing activity of mixed immune cells and single-cell populations.

To clarify the advantage of the mixed immune cells relative to single-cell populations, CD3^+^/CD8^+^ CTLs and CD3^+^/CD56^+^ NKT cells were separated from the mixed immune cells by cell sorting, and the killing activity of each population against Capan-1 and SW480 cells was evaluated. After the cell sorting, we cultured both CTLs and NKT cells for 3 days, by which time they returned to the same condition as the pre-sorted mixed immune cells. Consequently, compared with the negative control, the mixed immune cells, CD3^+^/CD8^+^ CTLs, and CD3^+^/CD56^+^ NKT cells all showed significant cytotoxicity against Capan-1 cells (Fig. 5A). Interestingly, the mixed immune cells exhibited significantly greater killing activity than CD3^+^/CD8^+^ CTLs and CD3^+^/CD56^+^ NKT cells (*p = 0.010, **p = 0.050, respectively). On the other hand, there was no significant difference in cytotoxicity between CD3^+^/CD8^+^ CTLs and CD3^+^/CD56^+^ NKT cells. Similarly, although the mixed immune cells, CD3^+^/CD8^+^ CTLs, and CD3^+^/CD56^+^ NKT cells all showed significant cytotoxicity against SW480 cells, the mixed immune cells exhibited greater killing activity than CD3^+^/CD8^+^ CTLs and CD3^+^/CD56^+^ NKT cells (Fig. 5B, ***p = 0.00048, **p = 0.021, respectively). There was also no difference in the cytotoxic effect of CD3^+^/CD8^+^ CTLs and CD3^+^/CD56^+^ NKT cells. These results indicate that the individual cell types in the mixed immune cell population interacted with each other and exerted potent synergistic cytotoxic effects.

## Discussion

ACT is a type of immunotherapy that was conceived long ago but has been actively investigated and improved since the development of ex vivo culture methods. In conventional ACT therapy, a patient’s peripheral blood is cultured ex vivo, and immune cell populations are expanded and then returned to the patient’s body. With the evolution of cell-engineering technology, this technique can now include genetic modification of immune cells before they are returned to the body. CAR T cells and CAR NK cells have demonstrated clinical benefit in the treatment of large B-cell lymphoma, acute lymphoblastic leukemia, multiple myeloma, non-Hodgkin’s lymphoma, and chronic lymphocytic leukemia^30–33^.

In the present study, we evaluated the feasibility of a novel culture method using both anti-CD3 mAb and anti-CD161 mAb for conventional ACT therapy. In contrast with typical culture methods for the expansion of LAK cells, CD3/CD161 co-stimulation results in increased numbers of a variety of immune cell populations. In particular, the number of CD3^+^/CD56^+^ NKT cells was increased 660-fold by the expansion, and these cells accounted for approximately 15% of the mixed immune cells. Transient CD3 stimulation is commonly used to effectively induce T-cell growth and activation. However, continuous CD3 stimulation mimicking chronic inflammation can lead to T-cell inactivation or apoptosis^34,35^. We found that like CD3 stimulation alone, transient CD3/CD161 co-stimulation promoted the growth and activation of mixed immune cells without obvious inhibitory effects.

NKT cells (which some studies refer to as NKT-like cells) have been described as a unique, CD1d-unrestricted T-cell population that differs from iNKT cells^36^. CD3^+^/CD56^+^ NKT cells, which possess both NK- and T-cell characteristics, can mediate non-MHC-restricted as well as MHC-restricted cell lysis, and several studies have reported their importance as cytotoxic effector cells in the immune response against cancer^37–40^. In this study, CD3^+^/CD56^+^ NKT cells showed stronger cytotoxic potential than CD3^+^/CD8^+^ CTLs, as indicated by their greater expression of granzyme B, IFN-γ, and TNF-α. However, simultaneously, they share the same MOAs; one is cell contact-dependent (direct) tumor cell killing via granzyme B, and another one is cell contact-independent (indirect) tumor cell killing via IFN-γ and TNF-α. The latter can occur via a bystander effect, whereby released cytotoxic cytokines cause damage after they are taken up by tumor cells. However, in this experiment, there were no differences between eightfold and 32-fold dilution of supernatant containing several cytotoxic cytokines such as IFN-γ and TNF-α. It seemed that the supernatant also contained some specific or non-specific cytokine-binding proteins, which may act as a buffer against the dilution method. Therefore, in further research we should evaluate these cytokines directly and quantitatively to validate the occurrence of cell contact-independent tumor killing and the bystander effect.

Non-MHC-restricted cell lysis by CD3^+^/CD56^+^ NKT cells may be effective against tumor cells that escape MHC-restricted cell lysis caused by CD3^+^/CD8^+^ CTLs. Therefore, CD3^+^/CD8^+^ CTLs and CD3^+^/CD56^+^ NKT cells can facilitate tumor cell damage cooperatively as a CTL-NKT circuit, in accordance with the bet-hedging strategy (Fig. 5C). Indeed, we found that the mixed immune cells had greater killing activity against cancer cell lines than CD3^+^/CD8^+^ CTLs or CD3^+^/CD56^+^ NKT cells alone. Because the mixed immune cells included a lower number of cytotoxic effector cells than single-cell populations of CD3^+^/CD8^+^ CTLs or CD3^+^/CD56^+^ NKTs, the mixed cells presumably possess synergistic cytotoxicity due to interactions between multiple immune cell populations, including CD3^+^/CD4^+^ helper T cells, CD3^+^/CD8^+^ CTLs, and CD3^+^/CD56^+^ NKT cells. In addition, other minor immune cell populations, such as CD3^+^/CD1d^+^ NKT cells, CD3^−^/CD56^+^ NK cells, CD3^+^/TCRγδ^+^ T cells, and CD3^-^/CD11c^+^/HLA-DR^+^ dendritic cells, might contribute to cytotoxicity in vitro despite accounting for smaller proportions (~ 1%) of the mixed immune cells. In previous reports, there were two types of mixed immune cells: (1) CIK cells, which are induced by anti-CD3 mAb and IL-2 after the induction of IFN-γ, and which consist of CD3^+^/CD56^+^ NKT cells, CD3^+^/CD8^+^ T cells, CD3^+^/CD4^+^ T cells, and CD3^−^/CD56^+^ NK cells^23^; and (2) a mixed immune cell population, which is induced by αGalCer-pulsed IL-2/GM-CSF and which consists of Vα24^+^/Vβ11^+^ iNKT cells, CD3^+^ T cells, and CD3^−^/CD56^+^ NK cells^24^. Interestingly, in the former group, CD3^+^/CD56^+^ NKT cells were predominant, as seen with our CD3/CD161 co-stimulation method. Moreover, they showed stronger cytotoxicity than LAK cells induced by CD3 stimulation and IL-2, without IFN-γ induction. On the other hand, in the latter group, Vα24^+^/Vβ11^+^ iNKT cells were predominant. This supports the concept of bet-hedging CTL-NKT or CTL-iNKT circuitry. Moreover, the mixed immune cells seem to retain a coordinated immune network.

In conclusion, we established a novel culture method using CD3/CD161 co-stimulation to effectively expand mixed immune cells. This combination of cells included both CD3^+^/CD8^+^ CTLs and CD3^+^/CD56^+^ NKT cells as two major cytotoxic effector populations. These mixed immune cells had a stronger cytotoxic effect than CD3^+^/CD8^+^ CTLs or CD3^+^/CD56^+^ NKT cells alone. We highlighted the concept of the bet-hedging CTL-NKT circuitry as the combined effect of CTL and NKT even if they have similar levels of cytotoxic activity. Moreover, other immune cells (CD3^+^/CD4^+^ helper T cells, CD3^+^/CD1d^+^ NKT cells, CD3^−^/CD56^+^ NK cells, CD3^+^/TCRγδ^+^ T cells, and CD3^−^/CD11c^+^/HLA-DR^+^ dendritic cells) may effectively cooperate to kill tumor cells. Further research is necessary to compare the molecular mechanisms underlying reconstituted immune networks, including CTL-NKT circuitry and anti-tumor activity, with those of CIK cells or αGalCer-pulsed IL-2/GM-CSF induced mixed immune cells.

Finally, CD3/CD161 co-stimulation may be a promising culture method to expand multiple, distinct immune cell populations. Clinical research is needed to investigate the efficacy of ACT using these cells.

## Data Availability

All data generated or analyzed during the current study are included in this article.
